# The role of Latin America and the Caribbean in global environmental health policy

**DOI:** 10.7189/jogh.13.03035

**Published:** 2023-12-08

**Authors:** Darren Dookeeram, Sandeep Maharaj, Steven De Jager, Mateo Losiewicz Cancela, Mariana Ceccon, Terrence Seemungal, Kareema Ali

**Affiliations:** 1 Faculty of Medical Sciences, University of the West Indies. St. Augustine, Trinidad and Tobago; 2 Faculty of Law and Political Science, Universitat Oberta de Catalunya, Barcelona, Spain.; 3 Planetary Health Alliance, Boston, Massachusetts, United States of America; 4 Samaritan’s Purse, Bogota D.C., Colombia; 5 Damos Leamos, B’nai B’rith, Uruguay; 6 United Nations News Editor, Rio de Janeiro, Brazil; 7 Eastern Regional Health Authority, Trinidad and Tobago

**Figure:**
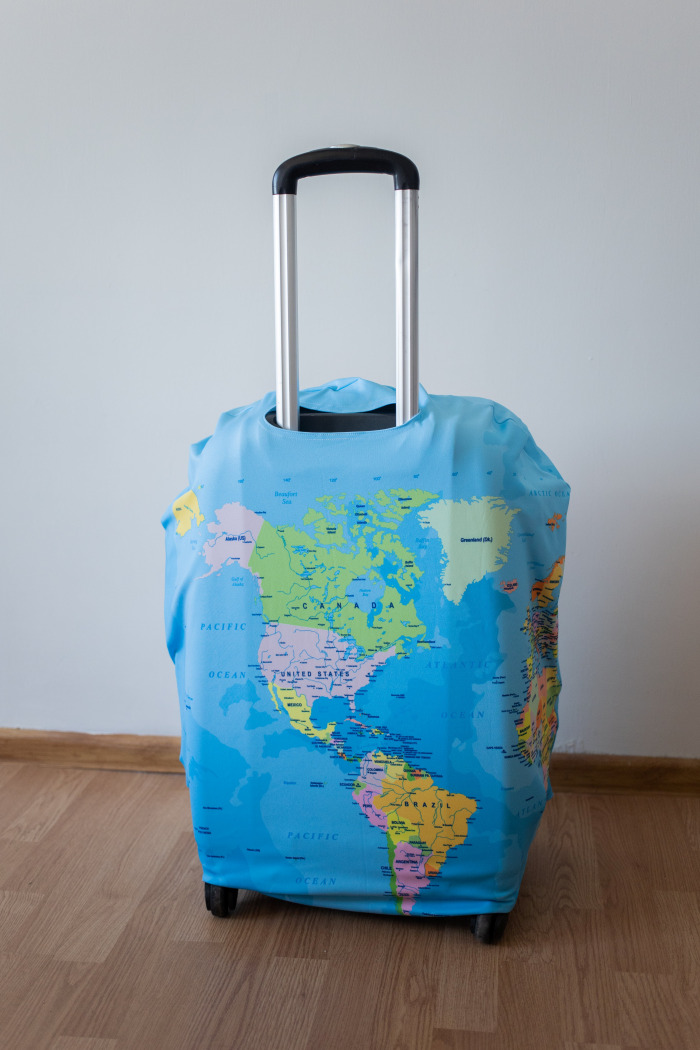
Photo: Suitcase with world map in room. Source: Created by Monstera Production, free to use under CC BY 4.0 license at Pixera. Available: https://www.pexels.com/photo/suitcase-with-world-map-in-room-7412055.

## SETTING A GLOBAL STAGE

The Latin America and the Caribbean (LAC) region encompasses a broad geographic expanse of states whose sociopolitical and economic systems have evolved over time as reflections of a variable colonial past, power imbalances with more advanced nation-states, and intra-regional cultural identity struggles. The varying extent of these “centre-periphery” internal and external factors in the post-Cold War era, coupled with the mechanisms of governance and policymaking, ultimately guide each state’s integration into an increasingly globalised world [1].

Despite the variety of nations within the region and their idiosyncratic differences, they are all similarly affected by the interlinked challenges of pollution, climate change, deforestation, carbon emissions, overpopulation, loss of biodiversity, resource depletion, and the resultant downstream disaster and public health concerns [2]. It is therefore imperative that regional and global solutions are devised that are acceptable and actionable to all international and regional stakeholders. Given the heterogenous global power structures, the literature suggests that regional governance of environmental issues may be more effective than international strategies to offset nuances in political governance structures and create buy-in [3]. This is particularly important given the volatile nature of countries driven by democratic presidentialism, in which policymaking changes with the ruling party’s leader and reflects their political will [4].

## THE ROLE OF LAC IN GLOBAL ENVIRONMENTAL HEALTH POLICY NEGOTIATIONS

LAC’s role in international environmental health protection negotiations can be considered analogous to its regional power imbalances, while the diverse process itself somewhat reflects its nationalistic sentiments. While some stakeholders, following the United Nations (UN) conference in Stockholm 1972, preferred to prioritise development and poverty reduction before environmental issues to the point of total inertia, the response after the Rio 1992 meeting became more active given the growing consensus that linked environmental health with human health and development.

The formation of the Group of 77/China (G-77/China) has given more commonality to a heterogeneous group of states who see themselves as underserved and allowed LAC countries to negotiate with the Global North for technical assistance in their environmental commitments. This has not prevented discord, however, as Mexico withdrew from the organisation, Brazil aligned with Russia, India, China and South Africa (the so-called BRICS), and the Bolivarian Alliance obstructed approval of the 2009 treaty in Copenhagen [5,6].

The role of non-state actors on environmental issues in LAC has been pivotal to introducing changes and progress. Externally funded international non-governmental organisations have been working with scientists in the region to advocate for change. However, this has proven challenging depending on the type of governance structure and has at times resulted in developed countries lobbying to to the World Bank to cut funding unless due diligence was given to environmental issues [5].

Despite these challenges, the Paris Climate Change Agreement has presented a significant buy-in from LAC. The Caribbean in particular, constituted by many small island developing states which are more vulnerable to environmental hazards, has advocated for change through its Caribbean community; in 2016, most nations in region acknowledged action plans to reduce carbon emissions per the Paris Agreement [7].

Achieving consensus in the region has been a notable challenge. Academia has mostly agreed on the importance of the environment and its protection as a key objective of states, regional bodies, and international organisations, with literature strongly suggesting that models of change centered around environmental preservation must include cost-benefit implementation strategies, so that any innovation does not disrupt populations and impose more challenges [8]. Consequently, the variable socio-political climate across the region has over time led to disputes on the issue of environmental governance. This is well exemplified by the contrast between the Bolivarian Alternative for the Americas (ALBA) and the Independent Association of Latin America and the Caribbean (AILAC). While the former seeks some extent of regionalism with its core ideology of rejecting policies which are derived from and primarily benefit the hegemony of the USA, the latter seeks integration which is conversely expanded more outwards into the southern hemisphere [6]. The attitude toward environmental governance through regional organisation is therefore predicated upon traditional foreign policy ideologies of groups of states within Latin America and the continuous struggle between the need for autonomy from and acquiescence to the hegemony of the USA [9]. However, the work of the AILAC has become more prominent than that of the ALBA as the world moves toward the Paris Agreement. While there might not be complete consensus, the Latin American region faces a high likelihood of significant downstream effects, despite being responsible for only 10% of carbon emissions, prompting a significant response in spite of hesitation from Bolivia, Venezuela and French Guiana [10,11]. Given the diversity of political interests of the region’s nations, creating buy-in for environmental governance requires the solidification of regionalism that is not threatened by external powers, meaning that, a regional body is more likely to effect change through creating trust than an international one, as AILAC has demonstrated to some extent.

The region’s economic challenges are thought to significantly influence policies on environmental health governance, as their economic implications are multidimensional and comprise both progressive and regressive elements. For example, the use of natural resources plays a pivotal role in the economies of most of the nation-states in Latin America. As the region’s foreign policy alignment shifted away from the USA because of their relative decline, while the highly consumptive Asian markets opened simultaneously, Latin America has used the opportunity to drive economic growth in response to social inequity [12]. This has become a proverbial double-edged sword from the environmental aspect, in that the growth of the natural resource sector is inherently accompanied by an acceleration of mining, deforestation, pollution, and creation of carbon sinks, making the process precariously imbalanced and requiring urgent need of regulation through treaties [13]. Such a regulation has been meaningfully achieved through the funding from international agencies, which align positive and negative financial interventions to government policies that similarly impact the environment [5]. Such policies have been continuously outlined at regional meetings of the United Nations Economic Commission for Latin America and the Caribbean since 1995, where shortcomings have been observed in the member-states’ ability to protect the environment, as have many instances in which they incentivised destructive behaviors [14]. The power asymmetry that has disadvantaged the Latin American region over the past decades has placed the states in a position where they seek to overcome the challenges through urbanisation and increasing free trade. While some aspects of policy can be regulated, these states must find a compromise to encourage population prosperity, growth, and development while being mindful of environmental issues [15]. However, such adherence is unlikely if policy directions emanate from international bodies who do not understand the nuances of the region, which is again suggestive of a need for a strong regional policy regulator with the capacity to assess and monitor progress.

There region’s diverse power structure comprises various actors with different levels of influence on environmental health governance policy, which can only be accurately deduced by considering conceptual and measurement frameworks of indicators. In 2020, the Inter-American Development Bank published composite data from ten countries which assessed context and resources, environmental governance, and environmental performance in ten key Latin American countries. While the report revealed heterogenous governance performance among the countries, the incompleteness of data sets across the region indicates difficulty in measuring influence. The study did identify Argentina, Bolivia, Brazil, Colombia, Costa Rica, Dominican Republic, El Salvador, Jamaica, Peru, and Uruguay as the first key actors [16]. Despite this, some key gross findings have consistently been noted in the literature on Latin America and environmental governance, such as Brazil’s considerable access to the Amazon reserves, large population, and important economic status. Other states such as Argentina, Mexico, and Colombia have also been highlighted as important actors due to their emerging economic status and political influence, as has Venezuela due its access to large hydrocarbon reserves [17]. This is coupled with the significant influence of non-state actors, concretised in the multi-stakeholder Marrakesh Partnership for Global Climate Action. In what Karin Bäckstrand termed as hybrid multilateralism in a 2017, such organisations have significant influence [18], the foremost being those acting within the United Nations Framework Convention on Climate Change system, which, on the national level, includes, activist groups, oil companies, professional unions, and associations and religious communities. Given this nation- and region-specific network, international organisations are dependent on regional governance which underlines the need for strengthening it to ensure environmental action.

## CONCLUSION AND THE WAY FORWARD

We believe that environmental health governance policy is at an important junction, where decisions are likely to affect human population health, socio-economic structures and environmental well-being. As such, it is imperative that LAC be prepared to have a formalised, unified approach to policy analysis and development, and that these considerations are mindful of other regions because of the interlocking effect of environmental change on the global level.

Given the diversity and complex multilateralism paired with the urgent need for environmental protection, new modes of governance should be considered to achieve actionable outcomes [19]. LAC countries are inserted into the international political economy with their own political, social, and cultural nuances, which is why each region requires unique strategic plans that may not be best understood by large transnational organisations. Consequently, it is imperative that the LAC bloc environmental governance structures should be strengthened at the regional level to foster positive change.
